# Prevalence, Risk Factors and Impact of Nutrition Interruptions in Critically Ill Children

**DOI:** 10.3390/nu15040855

**Published:** 2023-02-08

**Authors:** María José Solana, María Slocker, Zuriñe Martínez de Compañon, Marta Olmedilla, María Miñambres, Susana Reyes, Reyes Fernández, Eva Rodríguez, Silvia Redondo, Laura Díaz, María Sánchez, Jesús López-Herce

**Affiliations:** 1 Hospital General Universitario Gregorio Marañón, 28007 Madrid, Spain; 2 Servicio de Cuidados Intensivos Pediátricos, Hospital General Universitario Gregorio Marañón, Departamento de Salud Pública y Materno infantil, Universidad Complutense de Madrid, 28040 Madrid, Spain; 3 Primary Care Interventions to Prevent Maternal and Child Chronic Diseases of Perinatal and Development Origin Network (RICORS) RD21/0012/0011, Instituto de Salud Carlos III, 41092 Madrid, Spain; 4 Hospital Vall dHebrón, 08035 Barcelona, Spain; 5 Hospital Doce de Octubre, 28041 Madrid, Spain; 6 Hospital Clínico Universitario Virgen de la Arrixaca, 30120 Murcia, Spain; 7 Hospital Universitario Central de Asturias, 33011 Oviedo, Spain; 8 Hospital Universitario Nuestra Señora de la Candelaria, 38010 Tenerife, Spain; 9 Hospital Universitario de Cruces, 48903 Bilbao, Spain; 10 Complejo Hospitalario de Navarra, 31008 Pamplona, Spain; 11 Hospital Universitario Ramón y Cajal, 28034 Madrid, Spain; 12 Departamento de Salud Pública y Maternoinfantil, Facultad de Medicina, Universidad Complutense de Madrid, 28040 Madrid, Spain

**Keywords:** nutrition barriers, enteral nutrition, interruptions, PICU, critically ill children

## Abstract

**Background**: Enteral nutrition interruptions (ENI) are prevalent in the pediatric intensive care unit (PICU), but there is little evidence of their characteristics. **Methods**: This is a cross-sectional multicenter study including critically ill children on enteral nutrition. ENIs were classified as PICU procedures, procedures performed outside the PICU (PPOP), feeding intolerance and other criteria. The number and features of ENIs were collected. **Results**: A total of 75 children were enrolled. There were 41 interruptions affecting 37.3% of the patients with a median duration of 5 ± 9.4 h. The most common reason for ENI was PPOP (41.5%), followed by other criteria. Interruptions were considered preventable in 24.4% of the cases, but only eight were compensated. ENIs were more prevalent among children with cardiac disease (*p* = 0.047), higher PRISM (*p* = 0.047) and longer PICU stay (*p* = 0.035). There was association between PRISM and total interruption time (*p* = 0.02) and lower caloric intake (*p* = 0.035). Patients with respiratory illness (*p* = 0.022) and on noninvasive ventilation (*p* = 0,028) had fewer ENIs. ENI total time was associated with lower caloric (*p* = 0.001) and protein (*p* = 0.02) intake. **Conclusions**: ENIs are prevalent in PICU, especially in children with higher PRISM, longer PICU stays and cardiac disease, and result in lower caloric and protein intake.

## 1. Introduction

Malnutrition in critically ill children is common [1,2] and is associated with poor outcomes such as increased length of hospital stay, predisposition to infectious diseases and increased mortality and costs [1,3]. Many factors can contribute to undernourishment in this population. First, children have a lower percentage of lean and fat body mass, higher susceptibility to protein waste and higher resting energy expenditure, which increases the risk of acute malnutrition [4]. In addition, children are in a growth and development phase, with greater nutritional requirements than adults, which vary according to the stage of growth [4]. Those facts make children more vulnerable to fasting than adults. 

On the other hand, critical illness is characterized by severe, acute inflammation that favors protein catabolism and metabolic derangements, leading to malnutrition [5]. As sick children are particularly susceptible to protein depletion, the risk of developing malnutrition is even higher in these patients. 

There are other factors that may also contribute to the onset of malnutrition in critically ill children. For example, patients requiring mechanical ventilation or prolonged hospital stays, children under the age of two years and children with extensive burn injuries or congenital heart disease are high-risk groups for malnutrition [4,6].

Nutrition support is one of the key pillars to avoid undernourishment in critically ill children. Enteral nutrition (EN) is the preferred method of nutrition delivery to the critically ill child [2,6,7]. Among its many advantages are the following: it induces gastrointestinal mucosa trophism avoiding bacterial translocation, it is less expensive and it is associated with lower risk of infection than parenteral nutrition [2,8]. Early enteral nutrition (EEN), meaning enteral nutrition started within the first 48 h after pediatric critical care unit (PICU) admission, has been associated with higher caloric intake, fewer complications and lower costs [9,10,11,12] than delayed enteral nutrition (DEN). EEN has also been associated with a reduction in mortality, length of hospital stays and weight loss when compared to DEN [13]. A recent multicenter study in critically ill children also found that early enteral nutrition could be helpful in improving nutrient delivery, reducing time on mechanical ventilation and preventing constipation in sick children [14]; however, the presence of cardiac disease, mechanical ventilation and an age of over 12 months were risk factors associated with DEN. International guidelines recommend early enteral nutrition in critically ill children with hemodynamic support, extracorporeal life support and other situations once the child has been resuscitated [6,15], but enteral nutrition is often delayed due to gastrointestinal intolerance or the need to restrict fluid intake, making critically ill children prone to receiving insufficient calorie and protein intake [16].

However, delayed enteral nutrition is not the sole reason for low macronutrient intake; there are also other barriers that might interfere with nutrition delivery in critically ill children. Enteral nutrition interruptions (ENI) are highly prevalent in this population and may impact outcomes as they lead to discrepancies between caloric and protein prescription and delivery [15]. It is imperative to identify and characterize these barriers properly in order to provide the most suitable solution to compensate or avoid such interruptions, but there are few studies on this topic in critically ill children. 

Barriers for a well-established enteral nutrition have been classified in critically adult patients in four groups: process-related barriers, intensive care unit (ICU)-related interruptions, real or perceived feeding intolerance and ICU caregiver’s attitude and behavior [16]. This classification is important because depending on the barrier, different strategies to compensate the lost nutrition volume may be developed. 

Evidence regarding barriers for enteral nutrition in pediatric intensive care units is scarce and mainly based on surveys [17,18,19,20], reflecting the opinion of PICU caregivers but not necessarily showing the reality of PICU daily practice. Prospective studies to confirm the real prevalence of enteral nutrition interruptions and to analyze their features are necessary to raise awareness on this issue and to develop strategies aimed at preventing or compensating for their consequences. 

This study aims to provide insight on this matter, analyzing the prevalence of enteral nutrition interruptions in critically ill children admitted to the PICU, identifying risk factors and potentially avoidable barriers and analyzing the strategies to avoid or compensate for these barriers. 

## 2. Material and Methods

### 2.1. Study Design

This is a multi-cross-sectional multicenter study including critically ill children admitted to PICU for any reason. Ethics committee approval from the Institutional Review Board of the Hospital and at each participating site was obtained. Written informed consent was obtained from parents or legal guardians of the recruited patients.

PICUs were enrolled through the Nutrition and Digestive Pathology study group of the Spanish Society of Pediatric Intensive Care. To be eligible, sites were expected to have mixed (neonatal/pediatric) or pediatric intensive care units.

Critically ill children (1 month to 16 years) who were receiving enteral nutrition (EN) on the day of the study were included. Children not receiving EN or whose parents refused to participate in the study were excluded.

Three randomly chosen dates (23 March, 6 April and 20 April 2021) were analyzed to increase the number of enrolled patients. Data were collected over 24 h from 8:00 am of the selected day until 8:00 am of the following day to collect data from the three nursing shifts.

### 2.2. Data Collection

Medical staff recorded patient demographics, nutritional status, illness severity score (Pediatric Risk of Mortality III, PRISM III), diagnosis on admission, PICU length of stay and the amount and features of enteral nutrition interruptions. 

Nutritional assessment was also registered in every patient, including weight, length, body mass index (BMI) and BMI Z-score, calculated using an online tool (https://www.seghnp.org/ accessed on 3 December 2022) certificated by the Spanish Society of Pediatric Gastroenterology and Nutrition. A Z-score value ≤ −2 indicated undernourishment and a Z-score ≥ 2 indicated overweight/obesity.

The weight measurement was performed using a calibrated pan scale in infants if they had a favorable clinical condition or using the last registered weight if they were clinically unstable. Older children were weighted using a platform scale or bed scale if it was feasible, or otherwise using estimated weight.

Recumbent length measurement was obtained for patients 0–24 months using a solid length board, or infant meter, with the patient in a supine position. Height or stature was measured in patients older than 24 months who were able to stand using a vertical measuring tape fixed to a solid surface if patients were clinically stable (i.e., prior to a scheduled cardiac surgery). In clinically unstable children, the last measured height was recorded.

Different nutritional variables were also collected: route of enteral nutrition administration, prescribed caloric and protein requirements and delivered caloric and protein intake. Following international guidelines, energy requirements were calculated using the Schofield equation. Indirect calorimetry was available only in one participating site.

The need for inotropic support, noninvasive ventilation (NIV), mechanical ventilation (MV), ventricular assist device (VAD) or extracorporeal membrane oxygenation (ECMO) were also recorded.

Enteral nutrition interruption was defined as any EN cessation in the 24 h feeding plan indicated by the doctor or nurse either because of a procedure, real or perceived feeding intolerance or any unfavorable clinical condition.

Regarding enteral nutrition interruptions, the number, type of interruption, duration and actions aiming to compensate the lost nutrition volume during the interruption were analyzed. Compensation of the nutrition was defined as any action aiming to recover the lost volume of nutrition (i.e., increasing the nutrition rate).

The healthcare provider in charge identified avoidable enteral nutrition interruption episodes. These episodes were defined at the discretion of the researcher collecting the data as an unnecessary interruption of the enteral nutrition entailing a low risk of aspiration.

Enteral nutrition interruptions were classified into four different groups, as follows:

- The performance of invasive procedures in the pediatric intensive care unit (PICU procedures): tracheal intubation or extubation, venous or arterial catheter insertion, chest tube insertion or removal, wound care.
- The performance of invasive procedures outside the PICU: operating room, catheterization lab or diagnostic/therapeutic procedures in the radiology suite.
- Feeding intolerance that was defined by the presence of high gastric residual volume, vomiting, diarrhea, abdominal distension, abdominal pain or/and necrotizing enterocolitis.
- Other medical or caregiver criteria: hemodynamic instability, high inotropic support requirement, perceived high risk of aspiration, physical therapy, immunosuppressive therapy.

### 2.3. Statistical Analysis

SPSS statistical package version 25 (SPSS Inc., Chicago, IL, USA) was used for statistical analysis. Normal distribution of variables was tested with the Kolmogorov–Smirnov test. Continuous variables were expressed as median and interquartile range (IQR) and categorical variables as frequency and percentage. Chi-square (χ2) test was used for categorical variables, the Mann–Whitney test was used for continuous variables and the Rho of Spearman correlation index was used to assess the correlation between continuous variables. *p* values equal or less than 0.05 were considered statistically significant.

## 3. Results

### 3.1. Characteristics of the Study Population

The study population included 75 children admitted to nine PICU during the three study periods.

The median age of the group was 10 months (IQR 4–34), and half of them were male (53.3%). 

The most frequent diagnosis on admission was respiratory disease (49.3%), followed by cardiac disease (29.3%), neurological illness (6.7%), infectious disease (4%) digestive pathology (2.7%) and miscellanea (8%).

The median PRIMS III value on admission was 3 (IQR 0–10) and the mortality rate was 7.7%. The length of PICU stay was 32 days (IQR 8–67 days).

A large proportion of the patients were on mechanical ventilation (36%) or on noninvasive ventilation (37.3%). Thirteen children required inotropic support (17.3%), 34.7% required sedative drugs and 20% required opioids. 

Four patients (5.1%) were on continuous renal replacement therapy (CRRT), one child was on extracorporeal life support (ECMO) and five required a ventricular assistant device during the study period. 

Table 1 shows compared basal features between the patients who suffered enteral nutrition interruptions and those who did not. There was a statistically significant association between ENI and cardiac disease (*p* = 0.047) and between ENI and the risk of mortality at PICU admission measured by the PRIMS III score (*p* = 0.047). There was no association between ENI and age.

### 3.2. Nutritional Assessment

Median weight on admission was 8.53 kg (IQR 5–15 kg). A significant proportion of children (44.4%) were below the third percentile for the age group. According to BMI, 17.1% of the children were undernourished, of which 58.3% were severally undernourished. There was no statistically significant association between ENI and nutritional status or median weight.

### 3.3. Enteral Nutrition Route and Enteral Nutrition Interruptions 

There were forty-one interruptions during the study period that affected 37.3% of the patients. Twenty-eight children had one enteral nutrition interruption, ten children had two ENI and two patients had three interruptions during the study period.

The most common reasons for holding enteral nutrition were procedures performed outside the PICU (41.5%), followed by other medical criteria (36.5%). In the remaining patients, 9.8% of the interruptions were due to feeding intolerance and 12.2% were due to procedures performed within the PICU. The median duration of interruptions was 5 ± 9.4 h.

Staff responsible for interruptions were pediatric intensivists in 17 cases, nurses in 17 cases and anesthesiologists in 7 cases. Interruptions were deemed preventable by medical staff in 24.4% of the cases, and all of them were due to the performance of physical therapy or wound healing. 

Compensation of the lost nutritional volume was only performed in eight children and consisted of an increase in the nutrition rate to compensate the nutrition interruptions. Many of the compensation strategies were carried out within the administration of immunosuppressants. 

Transpyloric tube feeding was the preferred method for EN delivery (36%) in the study population, followed by gastric bolus (26.7%), continuous nasogastric tube (25.3%), gastrostomy tube (9.3%) and jejunal tube feeding (2.7%). Children on a transpyloric tube had more interruptions than those on nasogastric or gastrostomy tube (59.3% vs. 25%; *p* = 0.03). ENIs in children with a transpyloric tube were associated with a need for procedures performed outside the PICU (81.8% vs. 18.2%; *p* = 0.01).

Patients with respiratory illness and those on NIV had fewer ENIs (24.3% versus 50%; *p* = 0.022 and 78.6% vs. 21.4%; *p* = 0,028 respectively). On the other hand, interruptions were more prevalent among children with hemodynamic pathology (54.5% vs. 30.2%; *p* = 0.047) and those on mechanical ventilation (50% vs. 27.6%; *p* = 0.05). 

There was no association between ENIs and the need for MV, ECMO, ventricular assist devices or inotropic drugs, sedatives or opioid requirements. 

ENIs were associated with a higher PRISM score on admission (median PRISM 8, IQR 1.2–12 versus median PRISM 3, IQR 0–9; *p* = 0.047). There was also a statistically significant association between PRISM score and total interruption time (*p* = 0.02).

Patients with longer PICU stays were also more likely to have interruptions (42.5 days IQR 7.7–147.5 versus 17 days IQR: 7–46.5; *p* = 0.035). 

### 3.4. Enteral Nutrition Interruptions and Caloric and Protein Intake

The median prescribed energy intake was 73.6 kcal/kg/day (IQR 50.2–100.7 kcal/kg/day), and the median prescribed protein intake was 1.9 g/kg/day (IQR 1.3–2.7 g/kg/day).

ENI patients received a lower caloric intake than patients without ENIs (Table 2). The median energy intake was 51 kcal/kg/day in the group with nutrition interruptions and 70.6 kcal/kg/day in the group without ENIs, although the results did not reach statistical significance (*p* = 0.056). The median protein intake was also lower in children with ENIs than in the other group, although the results were not statistically significant (median protein intake 1.5 g/kg/day, IQR 0.75–2.3 versus 1.9 g/kg/day, IQR 1.1–2.6; *p* = 0.28) (Table 2).

However, there were statistically significant differences between prescribed and delivered caloric intake (*p* < 0.001) between both groups. There were also statistically significant differences between prescribed and delivered protein intake (*p* < 0.001) between children with and without ENIs.

ENI total time was also associated with lower caloric (*p* = 0.001) and protein (*p* = 0.02) intake. Children with higher PRISM score received lower energy delivery (*p* = 0.035).

## 4. Discussion

Enteral nutrition is the preferred method of nutrition delivery in critically ill children [2], but it is often interrupted for many reasons, leading to a lower energy and protein intake that contributes to malnutrition in critically ill children [4]. Previous surveys to determine the main reasons for enteral nutrition interruptions in critically ill adult and pediatric patients have been performed [17,18,19,21,22,23], but objective data about this important topic are scarce. It is imperative to know if these perceived barriers for enteral nutrition in critically ill children are actual barriers and to identify those children at high risk of nutrition interruptions.

Our study reveals that ENIs are prevalent in the PICU and may affect a considerable proportion of critically ill children. According to our data, up to 37.7% of our patients experience ENIs at least once during admission. However, as shown by the results of the present study, children may experience many nutrition interruptions during a single day. In fact, 30% of our patients experienced two or more feeding interruptions during the study period.

Our research also found certain risk factors for feeding interruptions that must be accounted for when managing enteral nutrition support in critically ill children. Patients with higher PRISM scores on admission and with longer PICU stays were at greater risk of withholding EN. Some of these results agree with previous reports [24], but unlike others such as Mehta’s results, our study showed that patients with higher PRISM scores suffered from more ENIs and longer total interruption time than those with lower PRISM values. These differences may be related to the greater need for invasive diagnostic or therapeutic procedures in the more severely ill patients. Furthermore, children with higher PRISM scores received lower energy delivery than those with lower scores, reflecting that sick patients are a high-risk group for malnutrition.

Our data are consistent with a previous study that found that ENIs were more prevalent among children with cardiac disease [25]. Fluid restriction has been identified as the main reason for nutrition interruptions in a prospective study conducted in one hundred and thirty-nine children with heart disease [26]. This report found that other factors such as surgery, longer length of PICU stay and longer feeding interruptions were also associated with lower energy intake in these patients, while a weight-for-age Z score of <−2 SD and a longer duration of mechanical ventilation were associated with lower protein intake [26]. Hemodynamic instability, which often occurs in critically ill children with cardiac disease, has been also described as one of the top three barriers to starting enteral feeding by a national survey distributed by the Saudi Critical Care Society [27]. Both the higher number of nutrition interruptions and the late enteral nutrition onset suggest that patients with hemodynamic pathology are at higher risk to develop or exacerbate undernourishment in PICU and should be closely monitored. However, this barrier may be potentially avoidable using some corrective strategies. First, PICU caregivers must have specific knowledge and skills regarding nutritional support for critically ill children with heart disease. Second, the use of protein- and energy-enriched nutritional formula in cases of fluid restriction could also prove helpful [28]. Finally, the use of protocols that lead the clinician to guide nutrition support may also be useful to avoid malnutrition in this population.

Noninvasive ventilation has been considered a barrier to enteral feeding because children requiring this type of respiratory support are at a higher risk of intubation [29]. However, recent evidence suggests that this type of respiratory support should no longer be a barrier to enteral feeding [21]. In our study, patients on NIV and with respiratory illness had fewer nutrition interruptions than children with other diagnoses and they did not develop more complications. On the contrary, patients on mechanical ventilation or fed by jejunal tube experienced more interruptions than children without mechanical ventilation or gastric feeding. Surprisingly, the use of transpyloric enteral nutrition was associated with more interruptions than the use of nasogastric or gastrostomy tubes. Transpyloric enteral nutrition has been used as an option to manage feeding intolerance in critically ill children or when there is a significant risk of aspiration [29]. A recent review revealed that jejunal tube feeding can reduce the residual gastric volume and enteral nutrition interruptions, thus increasing energy intake due to its ability to promote early nutrition and a rapid reach of targeted nutrition volume [29]. Our results may be explained by the higher number of procedures performed outside the PICU and the subsequent ENI experienced by transpyloric tube patients.

Many barriers leading to enteral nutrition interruptions have been suggested in critically ill patients, such as patient-related factors, feeding route factors and the feeding process [30]. In critically ill children, fluid restrictive policies [18,27], seriously ill children [18], the need for invasive procedures and operating room visits [18,20] and dietician-related issues [20,27] have also been identified as the main reasons to disrupt or not to start EN. Recognizing these barriers for enteral nutrition is imperative to improve feeding practices and to achieve optimal nutrition in the PICU [23]. 

In our study, the most common reason for holding enteral nutrition were procedures outside of the pediatric intensive care unit, which accounted for 41.5% of the total nutrition interruptions. This result confirms that of a previous worldwide survey that found that enteral feeds being withheld prior to surgery or other invasive procedures were one of the top three perceived barriers across PICU health caregivers [19]. There are some strategies that can be used to compensate nutrition interruptions due to invasive procedures, such as reducing fasting for diagnostic tests or invasive procedures, the use of small bowel feeding tubes when appropriate, or increasing the enteral nutrition infusion rate to provide the daily target volume [17].

The second cause of nutrition interruption in our study was the presence of other medical criteria, which accounted for 36.5% of ENIs. Interruption due to the administration of immunosuppressive drugs in solid organ transplant patients and those performing physical therapy were the most frequent reasons for withholding nutrition in this group. 

The third reason for enteral nutrition interruption in our study was the need to perform an invasive procedure within the pediatric intensive care unit (12.2% of the total nutrition interruptions). Procedures performed in critically ill children are important barriers for two reasons. First, most of these interruptions are most likely due to the clinician’s fear instead of the actual risk of the patient developing a complication [22], highlighting the need for educational programs in nutrition support for PICU caregivers. Second, there is no evidence available to guide which procedures should require fasting in critically ill children [31], so future research should be focused on identifying which therapies and procedures involve an actual risk of aspiration. 

The least prevalent reason for enteral nutrition interruptions was perceived or real feeding intolerance, which accounted for 9.8% of ENIs in our study. Feeding intolerance (FI) is a common problem in critically ill patients, resulting in nutrition interruptions. However, the lack of a consistent and validated definition for FI makes it difficult to know the real prevalence of this problem in sick patients and to obtain conclusive results on predictors and outcomes [32]. Gastric residual volume has been classically used as a marker of FI in critically ill patients [32], but recent clinical guidelines do not recommend its use on a routine basis in intensive care units because it does not reduce the risk of aspiration and pneumonia, but leads to unnecessary interruptions of the enteral nutrition that contribute to malnutrition [15]. However, despite these recommendations, a recent survey that included pediatric intensive care units from Spain and Latin America showed that this practice varies across the PICUs in terms of feeding intolerance interpretation, gastric residual volume threshold and management strategies [33]. This may be a limitation in the present study, since some researchers may consider gastric residual volume in different ways. Among the strategies proposed by Kozeniecki et al. to overcome this barrier is eliminating or reducing gastric residual volume measurement, considering short-term use of prokinetic agents, avoiding delayed initiation of enteral nutrition based solely on hypoactive bowel sounds or initiating parenteral nutrition in a timely manner based on both nutritional status and risk [17].

Previous surveys have also found delays and difficulties in obtaining small bowel feeding access, severe fluid restriction, no insufficient dietitian support during evenings, weekends and holidays, delays to preparing or obtaining nonstandard enteral feeds or feeding pump shortages in the unit as other potential barriers [20,28]. However, we did not explore these potential causes, so future prospective studies could focus on these barriers.

Achieving optimal nutrition in critically ill patients is essential, as inadequate nutrition is associated with organ dysfunction, length of stay and mortality. Previous reports have shown that a significant percentage of children do not receive adequate caloric and protein intake during PICU admission because of avoidable barriers [11,25,32,33,34,35]. One study performed in critically ill adults found that energy intake was as low as 33% of prescribed calories [36], while another reported that sick patients received approximately 60% of prescribed calories [37]. We found statistically significant differences between prescribed and delivered caloric and protein intake between both groups. Our data also showed that caloric and protein delivery were lower in children with nutrition interruptions than in children who did not have any feeding interruption. Median energy intake was 51 kcal/kg/day in the group with nutrition interruptions, while the group without ENIs received a median energy intake of 70.6 kcal/kg/day. We also found that median protein intake was lower in children with ENIs than in the other group with a median protein intake of 1.5 g/kg/day in the first group versus 1.9 g/kg/day in the second group, although this difference was not statistically significant. This means that patients who suffered nutrition interruptions received lower caloric and protein intake than children who did not have any interruption, highlighting the necessity of avoiding—or at least decreasing—the time of ENIs in critically ill children. 

Some studies have proven that enteral nutrition in critically ill patients is withheld for a mean of 4.8–7 h per day [38,39]. Feeding interruption time in our study was similar to previous research with a median duration of 5 ± 9.4 h. The total time of nutrition interruptions was also associated with lower total caloric and protein intake, highlighting the necessity of restarting enteral nutrition as soon as possible. 

Interruptions were considered preventable by medical staff in 24.4% of the cases, as most of them were performed during wound healing or physical therapy. However, strategies to compensate the missed volume were only used in 19.5% of the interruptions, even when most of them were considered unnecessary. Therefore, PICU caregivers should be trained in nutritional management, as this can affect factors that can reduce interruptions and increase energy and protein delivery [22].

Different strategies have been proposed to overcome these barriers in critically ill patients [16,40], such as defining the minimum time to hold EN before each interruption, the implementation of reduced fasting protocols and the compensation of the lost volume using volume-based feeding strategies [17]. Some examples of these strategies consist of dividing the daily nutrition target volume by 20 h instead of 24 h or prescribing enteral nutrition based on the total volume of formula to be delivered in 24 h and empowering the nurse to alter the rate of EN delivery to compensate for time off feeds [17]. In our study, only eight nutrition interruptions due to the immunosuppressive drug administration were compensated by an increase in the rate of the nutrition within the remaining time.

## 5. Conclusions

In summary, enteral nutrition interruptions are prevalent in critically ill children and result in lower caloric intake. Children with higher PRISM scores, longer PICU stays and mechanical ventilation, as well as those with cardiac disease, had more enteral nutrition interruptions than other children. PICU caregivers must be trained to recognize and prevent potentially avoidable interruptions and to develop strategies to compensate missed feeding volume. 

## 6. Limitations

The main limitation of our study is the small sample size, which limits the statistical power. Another major limitation is that this analysis has not considered potential confounders that may influence decision making about enteral nutrition interruptions. On the other hand, the lack of a consistent definition for feeding intolerance and the different interpretation and threshold of gastric residual volume and its management may also influence the results of the study. Therefore, larger studies are required to confirm these results.

## Figures and Tables

**Table 1:** Comparison of patients with any EN interruption versus those without ENI.

	Enteral Nutrition Interruption (*n* = 28)	No Enteral Nutrition Interruption (*n* = 47)	*p*-Value
Median age (months) (IQR)	15.5 (6.5–76.5)	7 (3–34)	0.19
Male gender (%)	39.3	61.7	0.06
Median weight (kg) (IQR)	10 (6.2–15.5)	7 (4.9–15)	0.174
Median height (cm) (IQR)	80 (65–111)	68 (56.3–87.7)	0.083
Median PRISM score (IQR)	8 (1.2–12)	3 (0–9)	0.047 *
Cardiac disease (%)	54	30.2	0.047 *
Respiratory illness (%)	24.3	50	0.022 *
Mortality (%)	0.14	0.04	0.18
Postpyloric feeds (%)	57.1	23.4	0.003 *
Mechanical ventilation (%)	50	27.6	0.05 *
Noninvasive ventilation (%)	78.6	21.4	0.028 *
Inotropic support (%)	25	12.7	0.21

IQR: interquartile range; EN: enteral nutrition; ENI: enteral nutrition interruption; *n*: number of patients. * Statistically significant value.

**Table 2:** Energy and protein delivery in children with any enteral nutrition interruption and without enteral nutrition interruptions.

	Enteral Nutrition Interruption (*n* = 28)	No Enteral Nutrition Interruption (*n* = 47)	*p* Value
Median delivered caloric intake (kcal/kg/day)	51 (20–92)	70.6 (50.5–101.2)	0.056
Median delivered protein intake (g/kg/day)	1.5 (0.75–2.3)	1.9 (1.1–2.6)	0.28
Difference between prescribed and delivered caloric intake (kcal/day)	62 (33–189.8)	0	<0.001 *
Difference between prescribed and delivered protein intake (g/day)	2 (0.8–7.9)	0	<0.001 *

* Statistically significant value.

## Data Availability

Data is unavailable due to privacy or ethical restrictions.
